# Health-related quality of life in patients with Kashin–Beck disease is lower than in those with osteoarthritis: a cross-sectional study

**DOI:** 10.1186/s13018-023-03803-8

**Published:** 2023-05-04

**Authors:** Zhankui Jin, Xueyuan Wu, Zhengming Sun, Ming Chen, Bo Yang, Xianghui Dong, Shizhang Liu, Yanhai Chang, Cuixiang Xu, Zhi Yi, Ming Ling

**Affiliations:** 1 Department of Orthopaedics, Shaanxi Provincial People’s Hospital, Xi’an , 710068 China; 2 Shaanxi Provincial Key Laboratory of Basic and Clinical Transformation of Bone and Joint Diseases, Shaanxi Provincial People’s Hospital, Xi’an, 710068 China; 3 Shaanxi Provincial Key Laboratory of Infection and Immune Diseases, Shaanxi Provincial People’s Hospital, Xi’an, China

**Keywords:** Kashin–Beck disease, Osteoarthritis, Endemic, Quality of life, Cross-sectional study, Questionnaire

## Abstract

**Background:**

Kashin–Beck disease (KBD) is an endemic deformable bone and joint disease, which affects the quality of life (QOL) of patients. We conducted a cross-sectional study of the QOL of KBD patients by a new KBD quality of life (KBDQOL) questionnaire.

**Methods:**

A total of 252 KBD patients and 248 OA patients came from Northwest China, and 260 healthy people living in the same area as KBD and osteoarthritis (OA) patients served as the controls. KBDQOL questionnaire was used to evaluate the QOL of all objects.

**Results:**

The average scores for physical functions, activity limitations, support of society, mental health and general health were significantly lower in KBD patients than that in OA patients and healthy people except for economics. Monofactor analysis showed that age, height, weight status, education level and grade of KBD had a significant effect on KBDQOL score. Multivariate analysis showed that grade of KBD was the influencing factor of physical function score; gender, age, height, grade of KBD and duration of symptoms were the influencing factors of activity restriction score; age and grade of KBD were factors affecting the general health score.

**Conclusion:**

The QOL of KBD patients was significantly lower than that of OA patients and healthy people. The KBDQOL questionnaire may be a promising tool for assessing the QOL of KBD patients.

**Supplementary Information:**

The online version contains supplementary material available at 10.1186/s13018-023-03803-8.

## Background

Although Kashin–Beck Disease (KBD) was reported more than 150 years ago, this disease continues to affect the health of some people in Asia [1]. KBD mainly affects the development of bone and cartilage of the limbs in children and adolescents from 5 to 15 years old [2, 3]. The primary lesions of KBD mainly invade the epiphyseal cartilage, epiphyseal plate cartilage and articular cartilage during the development of osteochondrosis of the limbs in children, and are characterized by multiple, symmetrical degeneration and coagulative necrosis of deep cartilage cells and their secondary osteoarthropathy [4].

The most common clinical symptoms of KBD include joint pain, joint thickening, joint deformity and limited mobility, severe short stature, short finger (toe) deformity, and muscle atrophy [5]. Compared with osteoarthritis (OA), the etiology of KBD is different from the occurrence and development mechanism of cartilage injury, but the clinical outcome is similar. KBD's knee joint deformity is significantly more serious than common OA, which seriously affects the patient's life and work. With the development of the disease, the deformity of the knee joint becomes more and more serious, and even the knee joint function is lost, which seriously affects the quality of life (QOL) of KBD patients [6]. Therefore, it is necessary to evaluate the quality of life and health status of KBD patients. However, no literature has reported the difference between the quality of life of KBD patients and OA patients.

There are already several methods for assessing the health status of patients with OA in different aspects. The Western Ontario and McMaster Universities Arthritis Index (WOMAC) and Lequesne indexes are special indicators of OA, which mainly focus on the effects of physical functions, physical symptoms and diseases, but they cannot reflect other aspects of QOL, such as psychology, social interactions, etc. [7, 8]. SF-36 and the European five-dimensional questionnaire (EuroQol five dimensions questionnaire, EQ-5D) have been widely used in the study of OA patients, but they are not very sensitive to measuring the results of specific disease interventions [9]. Most KBD patients cannot understand meaning of certain items in SF-36, because these patients come from remote mountainous areas, with low education and low comprehension skills. Therefore, Guo Xiong’s research team has developed a new, simple, and practical KBD Quality of Life (KBDQOL) questionnaire [9]. The questionnaire can capture the characteristics of KBD patients, can meet the psychometric characteristics required by clinical trials and observational research, and is easy to understand. In this study, we tried to use the KBDQOL questionnaire to assess the QOL and health status of KBD patients, and to understand the difference in QOL between KBD patients and local OA patients and healthy people.

## Methods

### Subjects

The subjects were divided into KBD patients, OA patients and a control group. The subjects were all from Shaanxi and Gansu provinces in China. The patients with knee pain were diagnosed as KBD or OA. The control group consisted of healthy people. All patients and healthy people were from the same KBD endemic regions of Shaanxi and Gansu provinces in China. None of the subjects had rheumatoid arthritis, tumors, stroke with limb inflexibility, severe cardiopulmonary disease, post-traumatic arthritis or deformity, persistent infection of the knee joint or other parts of the body, and other diseases that affect their lives quality.

### KBD clinical diagnosis

KBD was diagnosed as follows according to clinical criteria (GB16003-1995): A residence history of more than 6 months in a KBD endemic area, with symptoms such as multiple, symmetrical finger joint swelling or brachydactyly, etc. [10]. These diseases should be excluded, such as OA, rheumatoid arthritis, gout, rickets, cretinism, familial short stature, primary dwarfism, metaphyseal development disorders, achondroplasia, pseudoepiphyseal dysplasia, multiple epiphyseal dysplasia caused by short stature, mental retardation, and sexual development disorders. [11].

### Collection of baseline data

Baseline data of gender, age, height, weight, body mass index (BMI), education level, Kellgren–Lawrence (KL) classification, drinking, smoking, hypertension and diabetes in patients and the control group were collected.

The education level was divided into five standard levels:(1) no education completed, (2) first level (primary school), (3) secondary level (first phase), (4) secondary level (second phase), and (5) third level, which included university and other forms of higher education [12].

### KBDQOL evaluation

KBDQOL questionnaire was used to evaluate the QOL of KBD and OA patients. KBDQOL questionnaire is a QOL evaluation scale (Additional file 1: Table S1 [6]), and an important tool for evaluating QOL in KBD patients. KBDQOL data for each object was collected. The KBDQOL questionnaire has 28 items and 6 areas. Its brief contents are as follows: physical function (7 items), activity limitation (5 items), support of society (4 items), economics (3 items), mental health (5 items) and general health (four items). Each item has 5 options, and all items are scored from 1 to 5. The survey time is the past 4 weeks [9].

We calculate the average score for each domain as follows: average score for physical function = (Q1.1 + Q1.2 + Q1.3 + Q2.1 + Q2.2 + Q2.3 + Q3.4) / 7, average score for activity limitation = (Q1.4 + Q1. 5 + Q1.6 + Q1.7 + Q1.8) / 5, average support of society score = (Q4.2 + Q5.5 + Q5.6 + Q5.8) / 4, average economics score = (Q6.1 + Q6.2 + Q6.3) / 3, the average mental health score = (Q4.1 + Q4.4 + Q4.5 + Q4.6 + Q5.4) / 5, the total average general health score = (Q7. 1 + Q7.2 + Q7.3 + Q7.4)/4. The average score of each domain before and after surgery, 5 is the best average score [13]. A high score implies a high quality of life [9].

Investigator training: two investigators independently performed KBDQOL assessment of KBD patients to reduce observation bias. All investigators should receive 3 h of training. The purpose of the training is to understand the purpose and significance of the survey, the structure and definition of the questionnaire, the description and explanation of related knowledge, unify the meaning and filling method of the indicators, and clarify the survey workflow and precautions. The environment during the investigation should be a quiet environment, with no other people interfering with the investigation [13].

### Statistical analysis

Use SPSS (windows version 22.0) for statistical analysis. Demographic characteristics and clinical characteristics were expressed as mean ± standard deviation (measurement data) or absolute value (count data). The data of each group containing continuous variables were tested for normal distribution and homogeneity of variance. Two independent-sample t-tests were used for the comparison of continuous variables with normal distribution and homogeneity of variance, and single-factor analysis of variance was used for multi-group comparisons. Two groups of continuous variables without normal distribution and homogeneity of variance were compared using the Wilcoxon rank sum test, and Kruskal–Wallis test was used for multi-group comparison. The comparison of count data uses chi-square test. The reliability (Cronbach's alpha) and validity (exploratory factor analysis) of KBDQOL in patients with KBD and OA were analyzed. The gender, age, height, weight status, education and grade of KL were stratified to analyze the differences in KBDQOL scores between KBD patients, OA patients and healthy control. Because the dependent variables of physical function, activity limitation, support of society, economics, mental health, and general health data of patients were all non-normally distributed. The dependent variable was converted into categorical variable data, and the ordinal multi-category logistic regression analysis was used for multi-factor analysis. The dependent variable physical function, activity limitation, support of society, economics, mental health, and general health continuity variable data are converted into categorical variables, defined as: < 1.00 = 0, 1.00–1.99 = 1, 2.00–2.99 = 2, 3.00–3.99 = 3, 4.00–5.00 = 4. The independent variables gender, smoking, drinking, hypertension, and diabetes were designed as dummy variables in SPSS software. *P* value less than 0.05 is statistically significant.

## Results

### Demographic baseline data

**Fig. 1:**
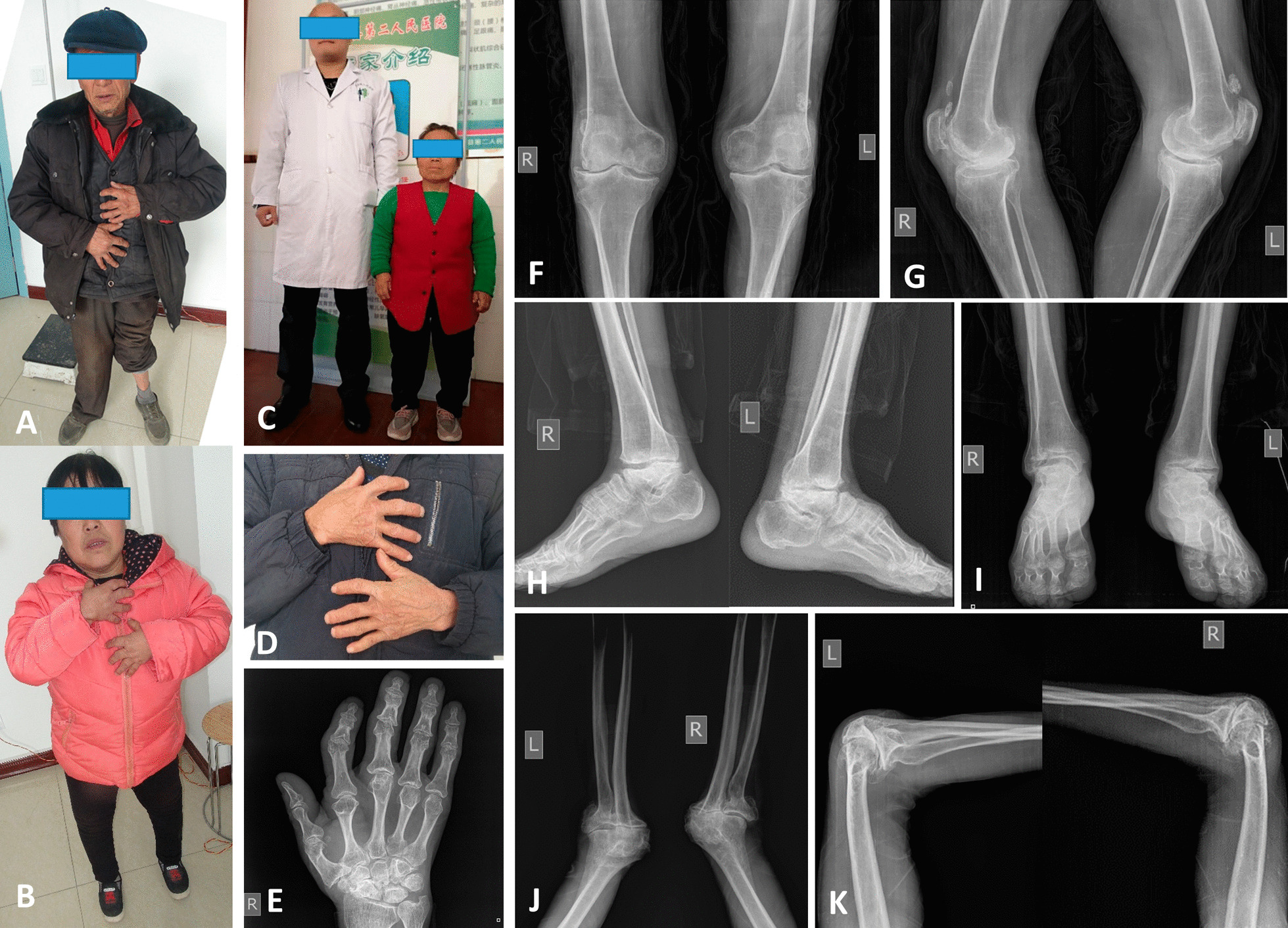
The physical characteristics and joint X-ray features of KBD patients. **A** The KBD patient presented with severe short deformity of the finger joint, osteophyte formation, and valgus deformity of knee joint. **B** This KBD patient presented with severe short finger joint deformity and knee varus deformity. **C** The KBD patient wearing a red coat shows shorter stature than a normal adult with a white coat. **D** A KBD patient shows a deformity of the finger joint. **E** X-ray of the hand of KBD patient showed thickening of the finger joints and osteophyte formation. **F** and **G** Radiographs of the knee joint in KBD patients showed joint space stenosis, arthrofacial osteosclerosis, and osteophyte formation. **H** and **I** Radiographs of the ankle in KBD patients showed narrowing of the ankle space, necrotic collapse of talus, and enlargement of the ankle joint. **J** and **K** Radiographs of the elbow in KBD patients showed valgus deformity, joint space narrowing, and osteophyte formation

There were 252 KBD patients, including 99 males and 153 females, with an average age of 59.94 ± 8.22 years old. There were 248 OA patients, including 105 males and 143 females, with an average age of 60.27 ± 8.07 years old. In the control group, there were 260 cases, including 118 males and 142 females, with an average age of 59.10 ± 8.60 years old. There was no statistical difference in demographic and clinical characteristics between KBD patients and OA patients and control group except height. The average height of KBD patients was significantly lower than that of OA patients and healthy people. The detailed data are shown in Table 1. The physical characteristics and joint X-ray features of KBD patients are shown in Fig. 1.

**Table 1:** Demographic characteristics and clinical features of the patients

	KBD patients (*n* = 252)	OA patients (n = 248)	Healthy people (n = 260)	P*
Gender, *n* (%)				0.337
Male	99 (39.29)	105(42.33)	118 (45.38)	
Female	153 (60.71)	143(57.66)	142 (54.62)	
Age (years, 95% confidence intervals)	59.94 ± 8.22 (58.92–60.96)	60.27 ± 8.07 (59.26–61.28)	59.10 ± 8.60 (58.05–60.15)	0.258
Age groups, years, *n* (%)				0.096
< 50	21 (8.33)	24(9.68)	33 (12.69)	
50–57	76 (30.16)	68(27.42)	68 (26.15)	
58–64	77 (30.56)	71(28.63)	96 (36.92)	
≧65	78 (30.95)	85(34.27)	63 (24.23)	
Height (m)	1.55 ± 0.09	1.62 ± 0.07	1.61 ± 0.07	< 0.001
Height groups, m, *n* (%)				< 0.001
< 1.50	59 (23.41)	11(4.44)	3 (1.15)	
1.50–1.59	104 (41.27)	84(33.87)	105 (40.38)	
1.60–1.69	80 (31.75)	121(48.78)	121 (46.54)	
≧1.70	9 (3.57)	32(12.90)	31 (11.92)	
BMI (kg/m^2^, 95% confidence intervals)	23.59 ± 3.35 (23.17–24.00)	23.72 ± 2.91 (22.89–23.68)	23.17 ± 2.99 (22.81–23.54)	0.141
BMI groups (kg/m^2^)				0.203
< 18.5	10 (3.97)	9(3.63)	5 (1.92)	
18.5–24.9	161 (63.89)	168(67.74)	192 (73.85)	
25.0–29.9	72 (28.57)	58(23.39)	55 (21.15)	
≧30.0	9 (3.57)	13(5.24)	8 (3.08)	
Educational level, *n* (%)				0.590
No education completed	87 (34.52)	82(32.06)	75 (28.85)	
First level (primary school)	76 (30.16)	74(29.84)	95 (36.54)	
Secondary level (first phase)	61 (24.21)	53(21.37)	58 (22.31)	
Secondary level (second phase)	26 (10.32)	35(14.11)	29 (11.15)	
Third level (university and other higher education)	2 (0.79)	4(1.61)	3 (1.15)	
Grade of KL, *n* (%)				0.518
Grade II	24 (9.52)	23(9.27)		
Grade III	134 (53.17)	144(58.06)		
Grade IV	94 (37.30)	81(32.66)		
Drinking, *n* (%)	31 (12.30)	38(15.32)	42 (16.15)	0.433
Smoking, *n* (%)	56 (22.22)	68(27.42)	61 (23.46)	0.368
Hypertension, *n* (%)	45 (17.86)	48(19.35)	51 (19.62)	0.862
Diabetes mellitus, *n* (%)	20 (7.94)	22(8.87)	23 (8.85)	0.913

^*^The samples did not have homogeneity of variance and were compared by a nonparametric rank sum test as the “Kruskal–Wallis test”. The comparison of count data uses chi-square test

### The KBDQOL scores in subjects

KBD patients on the KBDQOL questionnaire had an average of physical function score (2.43 ± 0.75), activity limitation score (3.60 ± 0.89), support of society score (3.23 ± 0.85), mental health score (3.23 ± 1.12), and general health score (2.49 ± 0.60), which was significantly lower than that in the OA patients (3.70 ± 0.68, 4.54 ± 0.49, 4.15 ± 0.49, 4.18 ± 0.44 and 2.60 ± 0.31) and control group (4.89 ± 0.14, 4.89 ± 0.20, 4.33 ± 0.50, 4.33 ± 0.38 and 3.73 ± 0.31).

**Fig. 2:**
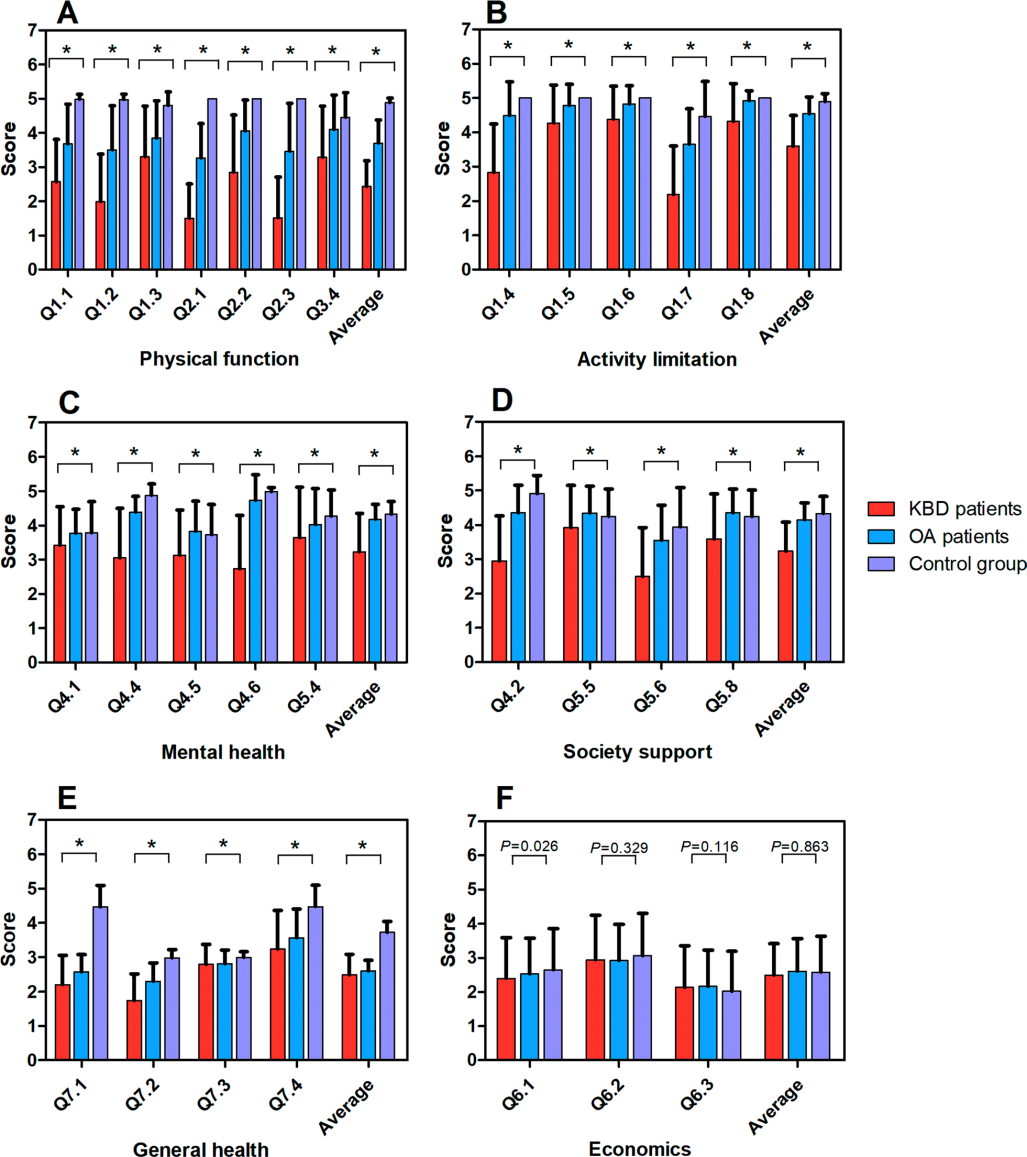
Comparison of KBDQOL scores between KBD patients and OA patients and control group. KBD patients on the KBDQOL questionnaire had an average score of physical function **A** activity limitation **B** mental health **C** support of society **D** and general health **F**, which was significantly lower than the average score of OA patients and the control group. The average score of KBD patients in economics **E** which was not statistically different from the OA patients control group, but on the average score of Q6.1 question **E** KBD patients were significantly lower than the control group

There was no statistical difference in the average score of economic between KBD patients (2.49 ± 0.92) and OA patients (2.61 ± 0.96) and healthy people (2.58 ± 1.05), but on the average score of Q6.1 question, KBD patients (2.40 ± 1.19) were significantly lower than the OA patients (2.53 ± 1.05) and control group (2.65 ± 1.20). The score comparison between KBD patients and OA patients and the control group on the 28 sub-items of the KBDQOL questionnaire is shown in Table 2 and Fig. 2.

**Table 2:** KBDQOL score comparison between KBD patients and OA patients and healthy people

Abbreviated item content of KBDQOL	Score (mean ± standard deviation, 95% confidence intervals)			P*
	KBD patients	OA patients	Healthy people	
Physical function	2.43 ± 0.75 (2.34–2.53)	3.70 ± 0.68 (3.61–3.79)	4.89 ± 0.14 (4.86–4.90)	< 0.001
Going up or down one step of stairs (Q1.1)	2.57 ± 1.24 (2.42–2.74)	3.68 ± 1.16 (3.53–3.82)	4.98 ± 0.15 (4.96–5.00)	< 0.001
Kneeling down (Q1.2)	1.99 ± 1.34 (1.82–2.18)	3.50 ± 1.29 (3.34–3.66)	4.97 ± 0.16 (4.95–4.99)	< 0.001
Bending down (Q1.3)	3.30 ± 1.49 (3.12–3.50)	3.85 ± 1.09 (3.71–3.99)	4.80 ± 0.40 (4.75–4.85)	< 0.001
Pain in joints (Q2.1)	1.50 ± 1.00 (1.37–1.63)	3.27 ± 1.00 (3.15–3.39)	5.00 ± 0.00 (5.0–5.0)	< 0.001
Duration of taking pain killer in days (Q2.2)	2.85 ± 1.67 (2.65–3.08)	4.06 ± 0.90 (3.95–4.17)	5.00 ± 0.00 (5.0–5.0)	< 0.001
Morning stiffness (Q2.3)	1.51 ± 1.20 (1.33–1.63)	3.46 ± 1.40 (3.29–3.64)	5.00 ± 0.00 (5.0–5.0)	< 0.001
Frequency of sleeplessness (Q3.4)	3.29 ± 1.50 (3.13–3.50)	4.10 ± 1.01 (3.97–4.23)	4.45 ± 0.73 (4.37–4.54)	< 0.001
Activity limitation	3.60 ± 0.89 (3.49–3.71)	4.54 ± 0.49 (4.47–4.60)	4.89 ± 0.20 (4.87–4.92)	< 0.001
Walking 1 km (Q1.4)	2.83 ± 1.44 (2.66–3.03)	4.49 ± 0.99 (4.37–4.61)	5.00 ± 0.00 (5.0–5.0)	< 0.001
Walking 100 m (Q1.5)	4.27 ± 1.11 (4.1–4.40)	4.78 ± 0.62 (4.70–4.86)	5.00 ± 0.00 (5.0–5.0)	< 0.001
Dressing yourself (Q1.6)	4.38 ± 0.97 (4.25–4.50)	4.83 ± 0.53 (4.76–4.90)	5.00 ± 0.00 (5.0–5.0)	< 0.001
Doing heavy labor such as farm work (Q1.7)	2.19 ± 1.42 (2.00–2.37)	3.65 ± 1.03 (3.52–3.78)	4.47 ± 1.02 (4.34–4.59)	< 0.001
Doing light labor such as cooking (Q1.8)	4.32 ± 1.10 (4.22–4.48)	4.92 ± 0.29 (4.88–4.96)	5.00 ± 0.00 (5.0–5.0)	< 0.001
Support of society	3.23 ± 0.85 (3.12–3.34)	4.15 ± 0.49 (4.08–4.21)	4.33 ± 0.50 (4.27–4.39)	< 0.001
Feel contribution to family duty (Q4.2)	2.95 ± 1.31 (2.78–3.12)	4.35 ± 0.81 (4.24–4.44)	4.91 ± 0.53 (4.84–4.97)	< 0.001
Feel supported by your family (Q5.5)	3.92 ± 1.22 (3.75–4.06)	4.34 ± 0.79 (2.25–4.45)	4.25 ± 0.80 (4.15–4.35)	0.002
Hang out, chat with neighbors (Q5.6)	2.50 ± 1.42 (2.33–2.69)	3.55 ± 1.02 (3.42–3.68)	3.94 ± 1.15 (3.80–4.08)	< 0.001
Have someone help you when you need (Q5.8)	3.58 ± 1.31 (3.41–3.74)	4.35 ± 0.70 (4.26–4.44)	4.24 ± 0.77 (4.14–4.33)	< 0.001
Economics	2.49 ± 0.92 (2.36–2.59)	2.61 ± 0.96 (2.49–2.73)	2.58 ± 1.05 (2.45–2.71)	0.863
Economy difficult (Q6.1)	2.40 ± 1.19 (2.22–2.52)	2.53 ± 1.05 (2.40–2.66)	2.65 ± 1.20 (2.51–4.80)	0.026
Borrow money (Q6.2)	2.94 ± 1.31 (2.76–3.09)	2.92 ± 1.06 (2.63–2.91)	3.06 ± 1.24 (2.91–3.21)	0.329
Can’t afford treating disease (Q6.3)	2.13 ± 1.23 (1.97–2.29)	2.16 ± 1.07 (2.41–2.65)	2.02 ± 1.17 (1.88–2.16)	0.116
Mental health	3.23 ± 1.12 (3.05–3.28)	4.18 ± 0.44 (4.12–4.23)	4.33 ± 0.38 (4.27–4.37)	< 0.001
Feel happy (Q4.1)	3.42 ± 1.13 (3.26–3.54)	3.77 ± 0.70 (3.67–3.85)	3.78 ± 0.91 (3.67–3.89)	< 0.001
Feel yourself is a burden to others (Q4.4)	3.05 ± 1.45 (2.84–3.20)	4.39 ± 0.46 (4.29–4.48)	4.87 ± 0.33 (4.83–4.91)	< 0.001
Feel blue mood (Q4.5)	3.13 ± 1.32 (2.93–3.26)	3.83 ± 0.88 (3.72–3.94)	3.72 ± 0.89 (3.61–3.83)	< 0.001
Feel embarrassed about bodily appearance (Q4.6)	2.73 ± 1.56 (2.51–2.91)	4.73 ± 0.75 (4.63–4.82)	4.98 ± 0.12 (4.96–5.00)	< 0.001
Feel that no one take care of you (Q5.4)	3.64 ± 1.47 (3.43–3.80)	4.03 ± 1.05 (3.90–4.16)	4.27 ± 0.77 (4.17–4.36)	0.001
General health	2.49 ± 0.60 (2.40–2.55)	2.60 ± 0.31 (2.55–2.64)	3.73 ± 0.31 (3.68–3.76)	< 0.001
In general, how about your health? (Q7.1)	2.20 ± 0.86 (2.08–2.30)	2.57 ± 0.51 (2.51–2.64)	4.47 ± 0.62 (4.38–4.54)	< 0.001
Compared to the same age and gender people, how about your health? (Q7.2)	1.74 ± 0.78 (1.61–1.81)	2.30 ± 0.53 (2.23–2.37)	2.97 ± 0.25 (2.95–3.00)	< 0.001
Compared to 1 month ago, how about your health? (Q7.3)	2.79 ± 0.59 (2.70–2.86)	2.81 ± 0.40 (2.75–2.86)	3.00 ± 0.16 (2.97–3.01)	< 0.001
In general, how satisfied are you with your quality of life (Q7.4)	3.24 ± 1.12 (3.08–3.37)	3.56 ± 0.85 (3.45–3.66)	4.47 ± 0.62 (4.39–4.55)	< 0.001
Reliability (Cronbach's alpha)				
Physical function (Q1.2, Q1.3, Q2.1, Q2.2, Q2.3, Q3.4)	0.823	0.809	0.831	
Activity limitation (Q1.4, Q1.5, Q1.6, Q1.7, Q1.8)	0.798	0.862	0.823	
Support of society (Q4.2, Q5.5, Q5.6, Q5.8)	0.847	0.856	0.853	
Economics (Q6.1, Q6.2, Q6.3)	0.789	0.913	0.846	
Mental health (Q4.1, Q4.4, Q4.5, Q4.6, Q5.4)	0.845	0.863	0.838	
General health In general, how about your health? (Q7.1, Q7.2, Q7.3, Q7.4)	0.853	0.864	0.813	
Validity	0.804	0.803	0.801	

^*^The samples did not have homogeneity of variance and were compared by a nonparametric rank sum test as the “Kruskal–Wallis test”

In order to reduce the influence of confounding factors, the gender, age, height, weight status, education and grade of KL were stratified to analyze the differences in KBDQOL scores between KBD patients, OA patients and healthy control (Tables 3, 4). We found no statistical difference in economics scores between groups. Except for the third level of education in general health, support of society and general health, Height greater than 1.7 m in support of society, the scores of physical function, activity limitation, support of society, mental health and general health were statistically different among the different groups.

**Table 3:** The stratified analysis of the physical function, activity limitation and support of society scores in subjects

Variable	Physical function				Activity limitation				Support of society			
	Score			*P*	Score			*P*	Score			*P*
	KBD	OA	Control		KBD	OA	Control		KBD	OA	Control	
Gender												
Male	2.43 ± 0.65	3.70 ± 0.64	4.95 ± 0.10	< 0.001	3.60 ± 0.87	4.60 ± 0.45	4.88 ± 0.21	< 0.001	3.21 ± 0.90	4.16 ± 0.52	4.38 ± 0.50	< 0.001
Female	2.43 ± 0.82	3.71 ± 0.72	4.83 ± 0.14	< 0.001	3.59 ± 0.91	4.49 ± 0.51	4.90 ± 0.19	< 0.001	3.24 ± 0.82	4.15 ± 0.46	4.30 ± 0.50	< 0.001
Age (years)												
< 50	2.26 ± 0.86	3.98 ± 0.64	4.90 ± 0.13	< 0.001	3.63 ± 0.95	4.65 ± 0.30	4.98 ± 0.14	< 0.001	3.18 ± 0.90	4.16 ± 0.42	4.23 ± 0.48	< 0.001
50–57	2.68 ± 0.72	3.83 ± 0.58	4.93 ± 0.10	< 0.001	3.99 ± 0.79	4.70 ± 0.33	5.00 ± 0.00	< 0.001	3.38 ± 0.95	4.20 ± 0.45	4.39 ± 0.45	< 0.001
58–64	2.41 ± 0.78	3.71 ± 0.62	4.45 ± 0.72	< 0.001	3.55 ± 0.84	4.56 ± 0.39	4.94 ± 0.15	< 0.001	3.21 ± 0.79	4.12 ± 0.50	4.32 ± 0.47	< 0.001
≧65	2.25 ± 0.69	3.52 ± 0.78	4.80 ± 0.16	< 0.001	3.25 ± 0.88	4.35 ± 0.63	4.67 ± 0.24	< 0.001	3.12 ± 0.79	4.13 ± 0.53	4.36 ± 0.60	< 0.001
Height (m)												
< 1.50	2.16 ± 0.65	3.91 ± 0.67	4.89 ± 0.14	< 0.001	3.31 ± 0.94	4.65 ± 0.42	4.95 ± 0.10	< 0.001	3.05 ± 0.72	4.13 ± 0.55	4.25 ± 0.35	< 0.001
1.50–1.59	2.53 ± 0.80	3.77 ± 0.64	4.84 ± 0.15	< 0.001	3.59 ± 0.89	4.57 ± 0.44	4.89 ± 0.22	< 0.001	3.27 ± 0.89	4.20 ± 0.44	4.35 ± 0.50	< 0.001
1.60–1.69	2.46 ± 0.75	3.69 ± 0.69	4.95 ± 0.11	< 0.001	3.72 ± 0.81	4.53 ± 0.52	4.94 ± 0.16	< 0.001	3.28 ± 0.85	4.12 ± 0.50	4.33 ± 0.61	< 0.001
≧1.70	2.79 ± 0.46	3.53 ± 0.76	4.87 ± 0.34	< 0.001	4.44 ± 0.53	4.41 ± 0.48	5.00 ± 0.00	< 0.001	3.50 ± 1.09	4.14 ± 0.52	4.31 ± 0.62	0.052
Weight status (by BMI, kg/m^2^)												
< 18.5	2.13 ± 0.44	3.35 ± 0.64	5.00 ± 0.00	< 0.001	3.40 ± 0.85	4.54 ± 0.40	4.92 ± 0.11	0.001	3.13 ± 0.85	3.64 ± 0.57	4.20 ± 0.37	0.041
18.5–24.9	2.46 ± 0.75	3.72 ± 1.14	4.88 ± 0.12	< 0.001	3.65 ± 0.83	4.51 ± 0.50	4.88 ± 0.21	< 0.001	3.25 ± 0.82	4.16 ± 0.48	4.32 ± 0.48	< 0.001
25.0–29.9	2.44 ± 0.80	3.73 ± 0.73	4.88 ± 0.17	< 0.001	3.61 ± 1.00	4.63 ± 0.46	4.88 ± 0.22	< 0.001	3.25 ± 0.89	4.14 ± 0.50	4.30 ± 0.58	< 0.001
≧30.0	2.03 ± 0.67	3.59 ± 0.71	4.73 ± 0.18	< 0.001	2.82 ± 0.74	4.35 ± 0.47	5.00 ± 0.00	< 0.001	2.86 ± 1.08	4.35 ± 0.35	4.59 ± 0.48	< 0.001
Education												
No education completed	2.18 ± 0.66	3.60 ± 0.76	4.91 ± 0.12	< 0.001	3.31 ± 0.89	4.40 ± 0.60	4.91 ± 0.18	< 0.001	3.08 ± 0.74	4.11 ± 0.44	4.35 ± 0.48	< 0.001
First level	2.45 ± 0.76	3.76 ± 0.66	4.89 ± 0.14	< 0.001	3.58 ± 0.90	4.58 ± 0.44	4.91 ± 0.18	< 0.001	3.09 ± 0.85	4.10 ± 0.46	4.34 ± 0.47	< 0.001
Secondary level (first phase)	2.66 ± 0.84	3.75 ± 0.62	4.84 ± 0.15	< 0.001	3.83 ± 0.83	4.63 ± 0.36	4.82 ± 0.25	< 0.001	3.49 ± 0.90	4.28 ± 0.47	4.29 ± 0.60	< 0.001
Secondary level (second phase)	2.57 ± 0.57	3.73 ± 0.66	4.91 ± 0.11	< 0.001	3.98 ± 0.73	4.60 ± 0.44	4.91 ± 0.22	< 0.001	3.40 ± 0.82	4.11 ± 0.61	4.32 ± 0.46	< 0.001
Third level	3.36 ± 0.71	3.82 ± 0.59	4.90 ± 0.08	0.054	4.50 ± 0.42	4.65 ± 0.30	5.00 ± 0.00	0.100	5.00 ± 0.00	4.63 ± 0.60	4.58 ± 0.14	0.187
Grade of KL												
Grade II	2.48 ± 0.84	4.47 ± 0.34		< 0.001	3.63 ± 0.85	4.85 ± 0.26		< 0.001	3.28 ± 0.79	4.10 ± 0.41		< 0.001
Grade III	2.53 ± 0.75	3.92 ± 0.35		< 0.001	3.76 ± 0.86	4.74 ± 0.20		< 0.001	3.29 ± 0.85	4.15 ± 0.49		< 0.001
Grade IV	2.26 ± 0.72	3.11 ± 0.76		< 0.001	3.35 ± 0.89	4.08 ± 0.58		< 0.001	3.14 ± 0.87	4.17 ± 0.50		< 0.001

*P* The samples did not have homogeneity of variance and were compared by a nonparametric rank sum test as the “Kruskal–Wallis test”. The comparison of count data uses chi-square test

**Table 4:** The stratified analysis of the economics, mental health and general health scores in subjects

Variable	Economics				Mental health				General health			
	Score			*P*	Score			*P*	Score			*P*
	KBD	OA	Control		KBD	OA	Control		KBD	OA	Control	
Gender												
Male	2.48 ± 0.90	2.61 ± 0.99	2.63 ± 1.06	0.734	3.35 ± 1.45	4.19 ± 0.46	4.38 ± 0.36	< 0.001	2.54 ± 0.61	2.88 ± 0.36	3.74 ± 0.30	< 0.001
Female	2.50 ± 0.94	2.61 ± 0.95	2.54 ± 1.05	0.674	3.15 ± 0.85	4.12 ± 0.42	4.28 ± 0.39	< 0.001	2.45 ± 0.58	2.76 ± 0.37	3.72 ± 0.32	< 0.001
Age (years)												
< 50	2.46 ± 1.08	2.67 ± 0.91	2.35 ± 0.98	0.415	3.01 ± 0.74	4.04 ± 0.42	4.25 ± 0.32	< 0.001	2.11 ± 0.78	2.96 ± 0.41	3.99 ± 0.10	< 0.001
50–57	2.45 ± 0.79	2.64 ± 1.02	2.51 ± 0.97	0.872	3.36 ± 0.92	4.20 ± 0.45	4.35 ± 0.43	< 0.001	2.48 ± 0.53	2.83 ± 0.37	3.93 ± 0.19	< 0.001
58–64	2.48 ± 1.03	2.67 ± 0.97	2.65 ± 1.13	0.456	2.96 ± 0.83	4.14 ± 0.44	4.33 ± 0.37	< 0.001	2.43 ± 0.56	2.82 ± 0.34	3.70 ± 0.27	< 0.001
≧65	2.55 ± 0.89	2.53 ± 0.94	2.67 ± 1.06	0.684	3.32 ± 0.92	4.15 ± 0.48	4.33 ± 0.37	< 0.001	2.65 ± 0.59	2.74 ± 0.36	3.42 ± 0.28	< 0.001
Height (m)												
< 1.50	2.34 ± 0.89	2.79 ± 1.15	2.33 ± 0.54	0.501	3.07 ± 0.76	4.24 ± 0.41	4.25 ± 0.44	< 0.001	2.29 ± 0.59	2.98 ± 0.44	3.81 ± 0.24	< 0.001
1.50–1.59	2.56 ± 0.85	2.51 ± 0.87	2.47 ± 1.00	0.507	3.33 ± 1.40	4.16 ± 0.44	4.32 ± 0.37	< 0.001	2.48 ± 0.61	2.81 ± 0.33	3.70 ± 0.32	< 0.001
1.60–1.69	2.51 ± 0.99	2.69 ± 1.02	2.72 ± 1.11	0.544	3.25 ± 0.96	4.13 ± 0.41	4.31 ± 0.37	< 0.001	2.60 ± 0.57	2.69 ± 1.02	3.76 ± 0.31	< 0.001
≧1.70	2.56 ± 1.26	2.53 ± 0.92	2.71 ± 1.13	0.836	3.02 ± 0.87	4.17 ± 0.54	4.23 ± 0.88	< 0.001	2.86 ± 0.33	2.71 ± 0.43	3.77 ± 0.31	< 0.001
Weight status (by BMI, kg/m^2^)												
< 18.5	2.60 ± 0.58	3.05 ± 0.83	3.07 ± 0.89	0.306	3.28 ± 1.06	3.97 ± 0.27	4.44 ± 0.43	0.060	2.50 ± 0.62	3.60 ± 0.22	2.68 ± 0.28	0.003
18.5–24.9	2.48 ± 0.92	2.63 ± 0.97	2.52 ± 1.01	0.648	3.17 ± 0.89	4.14 ± 0.43	4.28 ± 0.38	< 0.001	2.53 ± 0.54	2.81 ± 0.36	3.71 ± 0.33	< 0.001
25.0–29.9	2.50 ± 0.99	2.58 ± 0.99	2.78 ± 1.17	0.338	3.28 ± 0.91	4.15 ± 0.47	4.35 ± 0.39	< 0.001	2.48 ± 0.68	2.85 ± 0.38	3.68 ± 0.30	< 0.001
≧30.0	2.44 ± 0.85	2.41 ± 0.84	1.96 ± 0.77	< 0.001	2.82 ± 0.49	4.29 ± 0.43	4.48 ± 0.10	< 0.001	1.78 ± 0.48	2.63 ± 0.39	3.88 ± 0.23	< 0.001
Education												
No education completed	2.42 ± 0.91	2.59 ± 1.00	2.72 ± 1.04	0.241	3.07 ± 0.81	4.11 ± 0.46	4.33 ± 0.38		2.42 ± 0.59	2.79 ± 0.37	3.67 ± 0.30	< 0.001
First level	2.43 ± 0.79	2.61 ± 0.93	2.62 ± 1.03	0.539	3.28 ± 1.56	4.15 ± 0.45	4.31 ± 0.34		2.42 ± 0.67	2.8 ± 0.37	3.78 ± 0.29	< 0.001
Secondary level (first phase)	2.60 ± 0.99	2.68 ± 0.95	2.39 ± 0.60	0.149	3.27 ± 0.95	4.17 ± 0.41	4.36 ± 0.41		2.60 ± 0.52	2.82 ± 0.34	3.65 ± 0.37	< 0.001
Secondary level (second phase)	2.62 ± 1.04	2.55 ± 0.95	2.48 ± 1.05	0.708	3.45 ± 0.88	4.15 ± 0.43	4.31 ± 0.43		2.61 ± 0.49	2.83 ± 0.37	3.81 ± 0.25	< 0.001
Third level	3.67 ± 1.89	2.75 ± 1.50	2.44 ± 1.17	0.485	4.30 ± 0.42	4.45 ± 0.41	4.33 ± 0.31	0.968	3.13 ± 0.88	2.88 ± 0.60	4.00 ± 0.00	0.053
Grade of KL												
Grade II	2.90 ± 0.88	2.67 ± 1.24		0.273	3.23 ± 0.73	4.18 ± 0.39		< 0.001	2.54 ± 0.50	3.35 ± 0.16		< 0.001
Grade III	2.54 ± 0.98	2.61 ± 0.92		0.780	3.24 ± 0.85	4.17 ± 0.44		< 0.001	2.67 ± 0.48	2.86 ± 0.30		< 0.001
Grade IV	2.32 ± 0.81	2.60 ± 0.96		0.139	3.21 ± 1.50	4.09 ± 0.44		< 0.001	2.22 ± 0.67	2.57 ± 0.32		< 0.001

P, The samples did not have homogeneity of variance and were compared by a nonparametric rank sum test as the “Kruskal–Wallis test”. The comparison of count data uses chi-square test

### The monofactor analysis of the KBDQOL scores in patients with KBD

We performed a monofactor analysis of the KBDQOL scores for subjects to understand whether gender, age, height, weight status, education and grade of KL affect the KBDQOL scores for subjects. We found that physical function, activity limitation, mental health and general health were statistically different in different age groups. Physical function, activity limitation and general health were statistically different in different height groups. Activity limitation and general health had statistical differences between different weight status groups. Physical function, activity limitation and support of society had statistical differences in different education groups. Physical function, activity limitation, economics and general health have statistical differences in different grade of KBD groups. In summary, monofactor analysis showed that age, height, weight status, education and grade of KBD had statistically significant effects on the KBDQOL scores of KBD patients, while factors such as sex, disease duration, smoking, drinking, blood pressure, and blood glucose did not have a substantial effect on the KBDQOL scores of KBD patients (Table 5).

**Table 5:** The monofactor analysis of the KBDQOL scores in patients with KBD

Variable	Physical function		Activity limitation		Support of society		Economics		Mental health		General health	
	Score	*P*	Score	*P*	Score	*P*	Score	*P*	Score	*P*	Score	*P*
Sex		0.706		0.886		0.548		0.894		0.405		0.134
Male	2.43 ± 0.65		3.60 ± 0.87		3.21 ± 0.90		2.48 ± 0.90		3.35 ± 1.45		2.54 ± 0.61	
Female	2.43 ± 0.82		3.59 ± 0.91		3.24 ± 0.82		2.50 ± 0.94		3.15 ± 0.85		2.45 ± 0.58	
Age (years)		0.001		< 0.001#		0.229		0.961		0.010		0.007*
< 50	2.26 ± 0.86		3.63 ± 0.95		3.18 ± 0.90		2.46 ± 1.08		3.01 ± 0.74		2.11 ± 0.78	
50–57	2.68 ± 0.72		3.99 ± 0.79		3.38 ± 0.95		2.45 ± 0.79		3.36 ± 0.92		2.48 ± 0.53	
58–64	2.41 ± 0.78		3.55 ± 0.84		3.21 ± 0.79		2.48 ± 1.03		2.96 ± 0.83		2.43 ± 0.56	
≧65	2.25 ± 0.69		3.25 ± 0.88		3.12 ± 0.79		2.55 ± 0.89		3.43 ± 1.53		2.65 ± 0.59	
Height (m)		0.007		0.002		0.348		0.430		0.533		0.004
< 1.50	2.16 ± 0.65		3.31 ± 0.94		3.05 ± 0.72		2.34 ± 0.89		3.07 ± 0.76		2.29 ± 0.59	
1.50–1.59	2.53 ± 0.80		3.59 ± 0.89		3.27 ± 0.89		2.56 ± 0.85		3.33 ± 1.40		2.48 ± 0.61	
1.60–1.69	2.46 ± 0.75		3.72 ± 0.81		3.28 ± 0.85		2.51 ± 0.99		3.25 ± 0.96		2.6 ± 0.57	
≧1.70	2.79 ± 0.46		4.44 ± 0.53		3.50 ± 1.09		2.56 ± 1.26		3.02 ± 0.87		2.86 ± 0.33	
Weight status (by BMI, kg/m^2^)		0.251		0.048		0.811		0.934		0.419		0.009
< 18.5	2.13 ± 0.44		3.40 ± 0.85		3.13 ± 0.85		2.60 ± 0.58		3.28 ± 1.06		2.50 ± 0.62	
18.5–24.9	2.46 ± 0.75		3.65 ± 0.83		3.25 ± 0.82		2.48 ± 0.92		3.23 ± 1.24		2.53 ± 0.54	
25.0–29.9	2.44 ± 0.80		3.61 ± 1.00		3.25 ± 0.89		2.50 ± 0.99		3.28 ± 0.91		2.48 ± 0.68	
≧30.0	2.03 ± 0.67		2.82 ± 0.74		2.86 ± 1.08		2.44 ± 0.85		2.82 ± 0.49		1.78 ± 0.48	
Education		0.001		0.001		0.004		0.431		0.142		0.181*
No education completed	2.18 ± 0.66		3.31 ± 0.89		3.08 ± 0.74		2.42 ± 0.91		3.07 ± 0.81		2.42 ± 0.59	
First level	2.45 ± 0.76		3.58 ± 0.90		3.09 ± 0.85		2.43 ± 0.79		3.28 ± 1.56		2.42 ± 0.67	
Secondary level (first phase)	2.66 ± 0.84		3.83 ± 0.83		3.49 ± 0.90		2.60 ± 0.99		3.27 ± 0.95		2.60 ± 0.52	
Secondary level (second phase)	2.57 ± 0.57		3.98 ± 0.73		3.40 ± 0.82		2.62 ± 1.04		3.45 ± 0.88		2.61 ± 0.49	
Third level	3.36 ± 0.71		4.50 ± 0.42		5.00 ± 0.00		3.67 ± 1.89		4.30 ± 0.42		3.13 ± 0.88	
Grade of KBD		0.031		0.007		0.567		0.014		0.507		< 0.001*
Grade I	2.48 ± 0.84		3.63 ± 0.85		3.28 ± 0.79		2.90 ± 0.88		3.23 ± 0.73		2.54 ± 0.50	
Grade II	2.53 ± 0.75		3.76 ± 0.86		3.29 ± 0.85		2.54 ± 0.98		3.24 ± 0.85		2.67 ± 0.48	
Grade III	2.26 ± 0.72		3.35 ± 0.89		3.14 ± 0.87		2.32 ± 0.81		3.21 ± 1.50		2.22 ± 0.67	
Duration of symptoms (years)		0.777		0.522		0.799		0.667		0.136		0.332
< 10	2.55 ± 0.91		3.43 ± 0.92		3.18 ± 0.78		2.35 ± 1.00		3.26 ± 0.82		2.60 ± 0.51	
10–29	2.45 ± 0.71		3.67 ± 0.84		3.27 ± 0.84		2.57 ± 0.93		3.31 ± 1.43		2.50 ± 0.60	
30–44	2.37 ± 0.73		3.60 ± 0.88		3.17 ± 0.87		2.47 ± 0.90		3.04 ± 0.84		2.39 ± 0.60	
≧45	2.39 ± 0.80		3.53 ± 1.01		3.32 ± 0.90		2.47 ± 0.90		3.45 ± 1.00		2.57 ± 0.64	
Drinking		0.149		0.132		0.311		0.995		0.785		0.141
Absent	2.41 ± 0.78		3.56 ± 0.90		3.21 ± 0.84		2.49 ± 0.92		3.24 ± 1.14		2.46 ± 0.59	
Present	2.55 ± 0.56		3.86 ± 0.76		3.39 ± 0.93		2.52 ± 0.97		3.18 ± 1.00		2.64 ± 0.60	
Smoking		0.829		0.437		0.605		0.902		0.796		0.604
Absent	2.44 ± 0.78		3.57 ± 0.90		3.24 ± 0.86		2.51 ± 0.95		3.20 ± 0.88		2.48 ± 0.60	
Present	2.39 ± 0.67		3.68 ± 0.85		3.20 ± 0.81		2.42 ± 0.81		3.34 ± 1.73		2.50 ± 0.59	
Blood pressure		0.646		0.601		0.199		0.530		0.472		0.154
Normotension	2.44 ± 0.76		3.59 ± 0.90		3.25 ± 0.84		2.51 ± 0.92		3.21 ± 0.91		2.51 ± 0.59	
Hypertension	2.38 ± 0.74		3.64 ± 0.88		3.13 ± 0.90		2.42 ± 0.94		3.31 ± 1.84		2.36 ± 0.60	
Blood sugar		0.717		0.486		0.949		0.220		0.633		0.396
Diabetes mellitus	2.43 ± 0.75		3.60 ± 0.90		3.23 ± 0.84		2.52 ± 0.92		3.23 ± 1.14		2.48 ± 0.60	
Normal blood sugar	2.44 ± 0.84		3.52 ± 0.74		3.29 ± 0.95		2.22 ± 0.85		3.22 ± 1.00		2.6 ± 0.50	

*P*, the samples did not have homogeneity of variance and were compared by a nonparametric rank sum test as the “Kruskal–Wallis test” except labeled “*”

^*^Mann–Whitney U test

^#^The samples had homogeneity of variance and normal distribution were compared by a one-way ANOVA

### Multivariate analysis of factors affecting the scores of KBDQOL scale in KBD patients

We used ordinal multi-category logistic regression analysis to control confounding factors. KBD patients’ physical function, activity limitation, support of society, economics, mental health, and general health average scores were converted into categorical variables. Ordinal multi-category logistic regression analysis was performed on the influencing factors such as gender, age, height, weight status, education, grade of KBD, disease course, smoking, drinking, blood pressure and blood sugar.

The results showed that grade of KBD was the influencing factor of physical function score; gender, age, height, grade of KBD and duration of symptoms were the influencing factors of activity restriction score; grade of KBD was the influencing factor of economic score; and age and grade of KBD were factors affecting the general health score (Table 6).

**Table 6:** Multivariate analysis for control of confounding variables

Variable	Physical function		Activity limitation		Support of society		Economics		Mental health		General health	
	*P*	Exp(B)	*P*	Exp(B)	*P*	Exp(B)	*P*	Exp(B)	*P*	Exp(B)	*P*	Exp(B)
Sex												
Male	0.130	0.555	< 0.001	0.225	0.631	0.836	0.760	0.893	0.094	1.867	0.792	0.898
Female		1		1		1				1		1
Age	0.363	0.992	< 0.001	0.973	0.493	0.989	0.114	1.026	0.520	1.010	0.007	1.049
Height	0.489	3.001	0.038	27.294	0.903	0.831	0.629	0.481	0.386	0.269	0.120	13.987
Weight status (by BMI)	0.172	0.948	0.088	0.936	0.550	0.978	0.980	1.001	0.989	0.999	0.940	0.868
Education												
No education completed	0.385	0.279	0.999	2.423E-10	0.999	2.023E-10	0.086	0.085	0.999	2.551E-10	0.122	0.092
First level	0.675	0.545	0.999	3.764E-10	0.999	2.628E-10	0.123	0.113	0.999	3.033E-10	0.150	0.112
Secondary level (first phase)	0.821	0.721	0.999	6.012E-10	0.999	5.300E-10	0.204	0.166	0.999	3.723E-10	0.189	0.136
Secondary level (second phase)	0.847	0.754	0.999	6.826E-10	0.999	4.079E-10	0.215	0.171	0.999	4.931E-10	0.182	0.129
Third level		1		1		1		1		1		1
Grade of KBD												
Grade I	0.805	1.117	0.591	1.275	0.817	1.106	0.005	3.413	0.439	1.400	0.005	3.997
Grade II	0.007	2.067	0.001	2.502	0.473	1.203	0.179	1.410	0.322	1.282	< 0.001	5.635
Grade III		1		1		1		1		1		1
Duration of symptoms	0.239	0.990	0.023	1.020	0.811	1.002	0.498	0.995	0.196	0.990	0.180	0.988
Drinking												
Absent	0.692	0.845	0.069	0.398	0.572	0.790	0.557	1.272	0.371	1.448	0.360	0.663
Present		1		1		1		1		1		1
Smoking												
Absent	0.395	1.405	0.147	0.560	0.825	1.089	0.957	1.021	0.141	1.758	0.301	1.547
Present		1		1		1		1		1		
Blood pressure												
Normotension	0.849	1.063	0.626	0.854	0.377	1.312	0.655	1.146	0.588	1.180	0.112	1.716
Hypertension		1		1		1		1		1		1
Blood sugar												
Diabetes mellitus	0.797	1.127	0.942	1.035	0.778	1.134	0.213	1.743	0.869	1.076	0.734	0.849
Normal blood sugar		1		1		1		1		1		1

## Discussion

In this study, we conducted a large cross-sectional study to evaluate the QOL of patients with KBD by the KBDQOL questionnaire, a new KBD-specific QOL tool. The results emphasized that the QOL of KBD patients were significantly lower than that of OA patients and healthy people in the same region. Our data demonstrated that the KBDQOL questionnaire is a promising tool for assessing the QOL of KBD patients. To our knowledge, this study was the first assessment to examine differences in QOL between KBD patients and OA patients by a new quality of life instrument KBDQOL.

There are already many scales to evaluate different aspects of the health status of OA patients, such as WOMAC, Lequesne index, SF-36 and EQ-5D. Recently, the EQ-5D was used to measure the health-related quality of life (HRQOL) of KBD patients for the first time. Research results showed that KBD has a serious impact on patients' HRQOL, especially in pain, discomfort, mobility, anxiety, and depression [14, 15]. Compared with non-KBD participants and the general population in Beijing, KBD patients had a higher percentage of VAS scores and EQ-5D. The EQ-5D was used because there was no specific questionnaire that can be used to measure HRQOL related to KBD. KBD has some special characteristics, for example, its initial symptoms in early childhood are more serious than OA, so it is necessary to develop a dedicated HRQOL scale for KBD.

Most KBD patients are farmers living in rural areas of Shaanxi and Gansu Province and they have no primary education. KBDQOL is a low-educated KBD population survey that can be easily used in rural areas, and most of the questions are direct statements from KBD patients [9]. Some studies [16–18] reported the symptoms and signs of KBD, but none of the studies linked the clinical manifestations of KBD to patients’ disability, restriction of daily activities, the impact of disease on income, and the impact of disease on mood. We found that KBD patients do often have restricted activities, financial difficulties, and negative emotions. Physical disability leads to reduced income, affects family life, and further affects family relationships. Therefore, the final KBDQOL questionnaire includes items such as burdens, emotions, and financial difficulties related to daily activities.

Comparing KBDQOL with SF-36, some aspects are unique to KBDQOL (for example, social support, economics, etc.). Among the 12 physical function and activity limitation items of KBDQOL, only 66% and 50%, respectively, are part of the WOMAC and Lequesne indexes, respectively [18]. During the personal interview, more than 40% of the patients put forward two new contents as follow: Q2.2, in the past 30 days, "How many days do you need to take painkillers?" and Q3.4, "Do you sleep well? How many days are good?".

Because KBDQOL is a disease-specific QOL scale for KBD, it solves the problem of no scale model that can be used for comparison so far, and applies the World Health Organization QOL concept and framework to define the relevant items of KBDQOL [9]. EQ-5D is often used to measure the QOL of chronic musculoskeletal diseases and has good reliability [19]. In addition to social support and economics, the interrelationships of all aspects of KBDQOL are well related to the corresponding aspects of EQ-5D [14].

KBDQOL is suitable for KBD patients over 18 years old who have a clear awareness and ability to communicate [13]. Since more than 90% of KBD patients live in remote rural areas in China and have a low level of education [13], the content of the KBDQOL questionnaire is simple and easy to understand. It is suitable for face-to-face surveys for people educated in junior high school and below. Investigators should fill out the form and use a uniform standard description. For those who have a high school degree or above and can fill in themselves, they can fill out the form themselves.

During this investigation, there was a detail that cannot be ignored. When the investigator asked KBD patients "Do you feel that you are a burden or burden at home? (Q4.4)" and "Do you feel that you are not well-grown (short stature, deformed joints) and are unwilling to go out? (Q4. 6)", in these two questions, all patients answered: "always", and some patients showed very sad expressions when they answered the questions. When asked "Your physical condition is compared with people of the same age and sex (Q7.2)", all patients answered: "Much worse". All patients felt that they had brought a heavy burden to the whole family and had a stronger sense of inferiority compared with their peers. They were reluctant to go out due to their short stature, ugly appearance, or ugly walking posture. This reflected that KBD patients were under tremendous psychological pressure and their QOL is poor. It also demonstrated that KBDQOL can accurately capture the psychological characteristics of KBD patients.

Our study has potential limitations. Although the inductive method was used to explore the views of KBD patients on QOL, KBDQOL still lacks the views of other stakeholders, such as patients’ caregivers and family members. Therefore, further research is needed to test the clinical reactivity and applicability of KBDQOL in different cultural backgrounds.

## Conclusions

This study evaluated the QOL and health status of KBD patients by a new KBDQOL questionnaire. We conducted 28 questions in 6 domains including physical function, activity limitation, social support, economics, mental health, and general health for each object. The results highlighted that the average score of physical functions, activity limitations, support of society, mental health and general health of KBD patients was significantly lower than the average score of the OA patients and healthy people, except for economics. It substantially increases our knowledge in the field of health assessment of KBD patients. Therefore, we should pay more attention to how to improve the QOL of patients with KBD. KBD patients with mild pain can be relieved by non-surgical methods, such as oral pain reliever, Chinese herbal medicine, intraarticular injections of sodium hyaluronate, and physical therapy. KBD patients with severe pain can be relieved by surgical methods, such as arthroscopic debridement and joint replacement. These non-surgical and surgical approaches can improve the QOL of patients with KBD. Our data exhibited that the KBDQOL questionnaire may be a promising tool for assessing the QOL of KBD patients.

## Supplementary Information

**Additional file 1.** Details of KBDQOL questionnaire.

## Data Availability

The authors declare that the raw data are available and obtained through corresponding author Zhi Yi.

## Abbreviations

KBD: Kashin–Beck disease
OA: Osteoarthritis
QOL: Quality of life
EQ-5D: EuroQol five dimensions questionnaire
KBDQOL: KBD quality of life
HRQOL: Health-related quality of life
