# Supplementary material for: Health-related quality of life in patients with Kashin–Beck disease is lower than in those with osteoarthritis: a cross-sectional study
Source: J Orthop Surg Res. 2023 May 4;18:330. doi: 10.1186/s13018-023-03803-8 (PMC10161486; doi:10.1186/s13018-023-03803-8)
Supplement: Supplementary file 1 — Additional file 1. Details of KBDQOL questionnaire. [file 13018_2023_3803_MOESM1_ESM.docx]

**Supplementary Table 1** KBDQOL questionnaire

| Abbreviated item content of KBDQOL | | Alternative answer and score | | | | |
| --- | --- | --- | --- | --- | --- | --- |
|  |  | 1 | 2 | 3 | 4 | 5 |
| 1 | Going up or down one step of stairs (Q1.1) | absolutely can't | very difficult | medium difficulty | a little difficult | no difficulty |
| 2 | Kneeling down (Q1.2) | absolutely can't | very difficult | medium difficulty | a little difficult | no difficulty |
| 3 | Bending down (Q1.3) | absolutely can't | very difficult | medium difficulty | a little difficult | no difficulty |
| 4 | Walking 1 km (Q1.4) | absolutely can't | very difficult | medium difficulty | a little difficult | no difficulty |
| 5 | Walking 100 m (Q1.5) | absolutely can't | very difficult | medium difficulty | a little difficult | no difficulty |
| 6 | Dressing yourself (Q1.6) | absolutely can't | very difficult | medium difficulty | a little difficult | no difficulty |
| 7 | Doing heavy labor such as farm work (Q1.7) | absolutely can't | very difficult | medium difficulty | a little difficult | no difficulty |
| 8 | Doing light labor such as cooking (Q1.8) | absolutely can't | very difficult | medium difficulty | a little difficult | no difficulty |
| 9 | Pain in joints (Q2.1) | every day | 21d-29d | 11d-20d | 1d-10d | 0d |
| 10 | Duration of taking pain killer in days (Q2.2) | every day | 21d-29d | 11d-20d | 1d-10d | 0d |
| 11 | Morning stiffness (Q2.3) | every day | 21d-29d | 11d-20d | 1d-10d | 0d |
| 12 | Frequency of sleeplessness (Q3.4) | every day | 21d-29d | 11d-20d | 1d-10d | 0d |
| 13 | Feel happy (Q4.1) | always | often | sometimes | occasionally | no |
| 14 | Feel contribution to family duty (Q4.2) | always | often | sometimes | occasionally | no |
| 15 | Feel yourself is a burden to others (Q4.4) | always | often | sometimes | occasionally | no |
| 16 | Feel blue mood (Q4.5) | always | often | sometimes | occasionally | no |
| 17 | Feel embarrassed about bodily appearance (Q4.6) | always | often | sometimes | occasionally | no |
| 18 | Feel that no one take care of you (Q5.4) | always | often | sometimes | occasionally | no |
| 19 | Feel supported by your family (Q5.5) | always | often | sometimes | occasionally | no |
| 20 | Hang out, chat with neighbors (Q5.6) | always | often | sometimes | occasionally | no |
| 21 | Have someone help you when you need (Q5.8) | always | often | sometimes | occasionally | no |
| 22 | Economy difficult Q6.1 | always | often | sometimes | occasionally | no |
| 23 | Borrow money (Q6.2) | always | often | sometimes | occasionally | no |
| 24 | Can’t afford treating disease (Q6.3) | always | often | sometimes | occasionally | no |
| 25 | In general, how about your health? (Q7.1) | very poor | poor | just so so | good | very good |
| 26 | Compared to the same age and gender people, how about your health? (Q7.2) | much worse | worse | same | a little better | much better |
| 27 | Compared to 1 month ago, how about your health? (Q7.3) | much worse | worse | same | a little better | much better |
| 28 | In general, how satisfied are you with your quality of life (Q7.4) | very dissatisfied | not satisfied | just so so | satisfaction | very satisfied |
